# Utility of Facebook’s Social Connectedness Index in Modeling COVID-19 Spread: Exponential Random Graph Modeling Study

**DOI:** 10.2196/33617

**Published:** 2021-12-15

**Authors:** Beth Prusaczyk, Kathryn Pietka, Joshua M Landman, Douglas A Luke

**Affiliations:** 1 Center for Population Health Informatics Institute for Informatics Washington University School of Medicine in St. Louis Saint Louis, MO United States; 2 Department of Medicine Washington University School of Medicine in St. Louis Saint Louis, MO United States; 3 Truman State University Kirksville, MO United States; 4 Division of Computational and Data Sciences Washington University in St. Louis Saint Louis, MO United States; 5 Center for Public Health Systems Science Brown School Washington University in St. Louis Saint Louis, MO United States

**Keywords:** COVID-19, social media, social networks, network analysis, public health, utility, Facebook, connection, modeling, spread, United States, belief

## Abstract

**Background:**

The COVID-19 (the disease caused by the SARS-CoV-2 virus) pandemic has underscored the need for additional data, tools, and methods that can be used to combat emerging and existing public health concerns. Since March 2020, there has been substantial interest in using social media data to both understand and intervene in the pandemic. Researchers from many disciplines have recently found a relationship between COVID-19 and a new data set from Facebook called the Social Connectedness Index (SCI).

**Objective:**

Building off this work, we seek to use the SCI to examine how social similarity of Missouri counties could explain similarities of COVID-19 cases over time. Additionally, we aim to add to the body of literature on the utility of the SCI by using a novel modeling technique.

**Methods:**

In September 2020, we conducted this cross-sectional study using publicly available data to test the association between the SCI and COVID-19 spread in Missouri using exponential random graph models, which model relational data, and the outcome variable must be binary, representing the presence or absence of a relationship. In our model, this was the presence or absence of a highly correlated COVID-19 case count trajectory between two given counties in Missouri. Covariates included each county’s total population, percent rurality, and distance between each county pair.

**Results:**

We found that all covariates were significantly associated with two counties having highly correlated COVID-19 case count trajectories. As the log of a county’s total population increased, the odds of two counties having highly correlated COVID-19 case count trajectories increased by 66% (odds ratio [OR] 1.66, 95% CI 1.43-1.92). As the percent of a county classified as rural increased, the odds of two counties having highly correlated COVID-19 case count trajectories increased by 1% (OR 1.01, 95% CI 1.00-1.01). As the distance (in miles) between two counties increased, the odds of two counties having highly correlated COVID-19 case count trajectories decreased by 43% (OR 0.57, 95% CI 0.43-0.77). Lastly, as the log of the SCI between two Missouri counties increased, the odds of those two counties having highly correlated COVID-19 case count trajectories significantly increased by 17% (OR 1.17, 95% CI 1.09-1.26).

**Conclusions:**

These results could suggest that two counties with a greater likelihood of sharing Facebook friendships means residents of those counties have a higher likelihood of sharing similar belief systems, in particular as they relate to COVID-19 and public health practices. Another possibility is that the SCI is picking up travel or movement data among county residents. This suggests the SCI is capturing a unique phenomenon relevant to COVID-19 and that it may be worth adding to other COVID-19 models. Additional research is needed to better understand what the SCI is capturing practically and what it means for public health policies and prevention practices.

## Introduction

The COVID-19 (the disease caused by the virus SARS-CoV-2) pandemic has underscored the need for additional data, tools, and methods that can be used to combat emerging and existing public health concerns. Since March 2020, there has been substantial interest among researchers, public health professionals, infectious disease experts, and social media companies themselves in using social media data to both understand and intervene in the pandemic [1-7]. This is understandable given that nearly half of the world’s population (49% or 3.8 billion people) are social media users, with as many as 7 in 10 Americans reporting using at least one social media site.

One early example of using social media for novel purposes related to the pandemic was done by economists with expertise in modeling geographic and social data, who used a relatively new data set from Facebook called the Social Connectedness Index (SCI) to understand the spread of COVID-19 in the emerging hot spots of Italy and Westchester, New York [8]. The SCI is a measure of the strength of connectedness between two geographic areas as measured by Facebook friendships [9,10]. The researchers found that the SCI was associated with confirmed COVID-19 cases after controlling for geographic distance to the two early hot spots as well as income and population density [8].

Other researchers with backgrounds in economics, engineering, and management have also explored the utility of this data set as it relates to COVID-19. One group of researchers found that households in counties with relatively stronger social connections to early hot spots in China and Italy (as measured by the SCI) were more likely to comply with stay-at-home orders [11]. Others found that public health prevention practices that people in a given region adopt are significantly influenced by the policies and behaviors of people in other regions with whom there is a relatively strong SCI [12]. In other words, even between distant regions, the SCI was associated with people in those two regions having similar COVID-19–related behaviors, suggesting people are influenced by their social connections.

Building off this work, we sought to use the SCI to examine how social similarity of Missouri counties could explain similarities of COVID-19 cases over time. Additionally, we aimed to add to the body of literature on the utility of the SCI by using a novel modeling technique that allows for the modeling of relational data [13]. To our knowledge, this technique has not been used with the SCI, which is a relational data set, thus making it a highly relevant and appropriate method.

## Methods

### Study Design

In this cross-sectional study, we analyzed publicly available data to test the association between the SCI and COVID-19 spread in Missouri using exponential random graph models (ERGMs). This study was reviewed by the institutional review board and deemed nonhuman participant research.

### Data Sources

#### Social Connectedness Index

The SCI was obtained through the Facebook Data for Good program. The Facebook Data for Good program creates and makes available a variety of tools and data sets that are built from privacy-protected data from the Facebook platform and other publicly available data sources such as satellite imagery. Data sets in the program include the SCI, electrical distribution grid maps, the Inclusive Internet Index (a measure of internet accessibility), the Climate Change Survey, and more.

The SCI measures the relative probability of a Facebook friendship link between a given Facebook user in location A and a user in location B. It is calculated by dividing the number of Facebook friends between two locations divided by the number of Facebook users in location A multiplied by the number of users in location B. The SCI data set includes values for locations from the zip code level up to the country level and is an anonymized snapshot from a single point in time. The locations of Facebook users are assigned based on their information and activity on Facebook, including their public profile information as well as device and connection information.

The SCI is a single data set calculated based on Facebook friendships in March 2020; therefore, additional time points of the SCI could not be included in the model or in sensitivity analyses.

#### COVID-19 Data

To determine which Missouri counties had similar COVID-19 spread, we used data obtained from the Johns Hopkins University’s Coronavirus Resource Center. The data on United States COVID-19 cases and deaths made available through the Center are compiled by the Johns Hopkins Center for Systems Science and Engineering, which is updated daily. They retrieve all state data from their respective state departments of health or other local government reporting agency, and for Missouri, those sources are the Missouri Department of Health, St. Louis City Department of Health, St. Louis County Department of Health, and Nodaway County Health Center. We obtained daily new case counts for every county in Missouri starting on March 8, 2020 (the day the first case of COVID-19 was recorded in the state), through September 30, 2020, which was the time we conducted the analyses.

#### Population, Rurality, and Distance Data

Data on each county’s population and its rurality were obtained from the United States Census Bureau from the 2010 Census database [14]. Distance between each county pair was obtained from the 2020 TIGER/Line shapefiles, also available from the US Census Bureau [15].

### Analysis

All analyses were conducted using R version 4.0.3 (R Foundation for Statistical Computing) with the packages statnet and ergm. Alpha levels were set at .05.

#### Data Management

Every county pair has an SCI value, so this variable did not need to be computed, though this variable was log transformed.

To create a measure of two counties’ similarity in COVID-19 case counts, we used the daily new case counts as each county’s “trajectory” of COVID-19 and conducted a Pearson correlation test between each county’s trajectory. We then used a 0.60 correlation coefficient cutoff to classify each county pair as either having highly correlated COVID-19 case count trajectories or not. The 0.60 cutoff was chosen based on established recommendations [16]. This binary variable was our primary outcome.

The total county population was log transformed, and the distance between every county pair was calculated using the distance between the centroids of each county in the shapefiles. The percent of the county that was classified as rural was not computed or transformed before being entered into the analytical model.

We originally intended to include demographic characteristics of residents at the county level, but given the lack of diversity on characteristics such as age, race, and ethnicity across Missouri, including these data in the model caused it to not converge. Therefore, we were unfortunately unable to include them.

#### Modeling

Our basic modeling approach was to examine the relationship between the social media connections (as measured by the SCI) and COVID-19 case counts across Missouri counties. To do this, we used exponential random graph modeling.

ERGMs model relational data, and the outcome variable must be relational and binary, representing the presence or absence of a relationship. In our model, this was the presence or absence of a highly correlated COVID-19 case count trajectory between two given counties in Missouri. The model was built sequentially, starting with a null model. Next, all covariates except the SCI were entered into the model. Distance between every county pair was entered into the model as a relational term, meaning it represented a relationship between every county pair. Total county population and the percent of the county classified as rural were both entered into the model as object-level terms, meaning instead of the data representing a relationship between every county pair, these data were singular attributes of each county. After running this model, the SCI was entered into the last model and the Akaike information criterion (AIC) was used to compare overall model fit. Odds ratios (ORs) and 95% CIs are also reported.

## Results

### COVID-19 Case Count Trajectories

Missouri reported its first COVID-19 case on March 8, 2020, and at the time of analysis (September 30, 2020) the state had reported 129,733 cumulative cases with a 7-day average of 1127. Each county’s average daily new case count data are available in Multimedia Appendix 1.

Of the 6555 different county pairs ([115 counties * 114] / 2 = 6555), the range of correlations was –0.09 to 0.90 with an average correlation of 0.36. Of those, 1114 county pairs had COVID-19 case count trajectories correlated at 0.60 or greater. These 1114 county pairs then represented the relationship we predicted in the model.

### Exponential Random Graph Model

The results of the sequential model building process are presented in Table 1. In the final model, we assessed the likelihood that two counties in Missouri had highly correlated COVID-19 case count trajectories based on their level of social connectedness, controlling for the total population size of the counties, the percent of the counties that were rural, and the distance between the two counties. The model fit improved sequentially as evident by the decreasing AIC value as more covariates were entered into the model.

**Table 1:** Sequential building of an exponential random graph model of the relationship between the Social Connectedness Index (SCI) and Missouri counties’ similar COVID-19 case counts from March to September 2020.

		Null model^a^			Model 1^b^		Model 2^c,d^			
		b (SE)		*P* value	b (SE)	*P* value	b (SE)		OR^e^ (95% CI)	*P* value
Intercept		–2.40 (0.04)		<.001	–14.63 (0.12)	<.001	–14.85 (0.94)		0.00 (0.00-0.00)	<.001
Total county population (logged)		N/A^f^		N/A	0.44 (0.04)	<.001	0.51 (0.07)	1.66 (1.43-1.92)		<.001
Percent of county that is rural		N/A		N/A	0.01 (0.00)	.001	0.01 (0.00)	1.01 (1.00-1.01)		<.001
Distance in miles between county pairs		N/A		N/A	–0.62 (0.16)	<.001	–0.55 (0.15)	0.57 (0.43-0.77)		<.001
SCI^g^ (logged)		N/A		N/A	N/A	N/A	0.16 (0.04)	1.17 (1.09-1.26)		<.001
**Model fit**										
	Akaike information criterion	3251	N/A		2707	N/A	2691	N/A		N/A
	Bayesian information criterion	3773	N/A		2741	N/A	2731	N/A		N/A
	Log likelihood (*df*)	–1882.008 (1)	N/A		–1485.785 (4)	N/A	–1477.192 (5)	N/A		N/A

^a^Null model with no covariates.

^b^Model 1 included all covariates, except the SCI.

^c^Model 2 included all covariates, including the SCI.

^d^The geometrically weighted edgewise shared partner term *gwesp* was included in models 1 and 2.

^e^OR: odds ratio.

^f^N/A: not applicable.

^g^SCI: Social Connectedness Index.

All covariates were significantly associated with two counties having highly correlated COVID-19 case count trajectories. As the log of a county’s total population increased, the odds of two counties having highly correlated COVID-19 case count trajectories increased by 66% (OR 1.66, 95% CI 1.43-1.92). (Log scales are commonly used when examining population growth; it also is helpful here for comparing changes in ratios or proportions.) As the percent of a county classified as rural increased, the odds of two counties having highly correlated COVID-19 case count trajectories increased by 1% (OR 1.01, 95% CI 1.00-1.01). As the distance (in miles) between two counties increased, the odds of two counties having highly correlated COVID-19 case count trajectories decreased by 43% (OR 0.57, 95% CI 0.43-0.77). For our main outcome, we found that as the log of the SCI increased between two counties, the odds of those two counties having highly correlated COVID-19 case count trajectories increases by 17% (OR 1.17, 95% CI 1.09-1.26), controlling for the counties’ population size, rurality, and the distance between the two counties.

## Discussion

### Principal Findings

We found that as the likelihood of Facebook friendships between two counties increases, as measured with the SCI, the odds of those two counties having strong, positive correlations of their COVID-19 daily new case count trajectories also significantly increased. This relationship remained significant when controlling for the distance between the two counties, their rurality, and their total population sizes.

These results build upon and align with prior, preliminary research using the SCI to understand COVID-19 spread. [8,11,12] These results also confirm the “signal” in the SCI “noise,” meaning there is something uniquely captured in the SCI and Facebook friendships that cannot be explained by geography, distance, or population size.

The primary reasons for conducting this study were to assess if the relationship between the likelihood of Facebook friendships and COVID-19 spread could be explained by other factors. For example, it makes intuitive sense that two urban counties are more likely to have similar COVID-19 case count trajectories because, in general, urban areas had more cases earlier in the pandemic than rural areas [17]. It also makes sense that two urban counties would be more likely to share Facebook friendships than an urban and a rural county [18]. Likewise, it is reasonable to expect that two counties next to each other would be more likely to share Facebook friendships than two counties hundreds of miles apart [9]. Could the SCI signal as it relates to COVID-19 spread be explained by these other factors? Our results suggest there is something above and beyond these other factors that the SCI represents; however, it is not clear what exactly that is.

One possibility is that people tend to form friendships and social connections to those who share similar belief systems [12,19]. This could suggest that two counties with a greater likelihood of sharing Facebook friendships means residents of those counties have a higher likelihood of sharing similar belief systems, in particular as they relate to COVID-19 and public health practices. For example, perhaps residents of two counties with a relatively high SCI value are equally likely to wear masks or not, restrict travel or not, etc. Residents sharing similar public health practices could explain why counties with relatively high SCI values are also more likely to have similar COVID-19 case count trajectories. Similar results have been found in earlier studies using the SCI [12].

Another possibility is that the SCI is picking up travel or movement data among county residents. People are more likely to form Facebook friendships with people they have offline connections with, and these offline connections may stem from a physical location such as a school, place of worship, or place of employment [20,21]. Therefore, a resident of one county may have a lot of Facebook friends in a neighboring county because that resident works at a large business in that neighboring county and travels there multiple days a week. That resident may also frequent restaurants and other businesses near their place of employment, increasing the opportunities to form friendships in this neighboring county. In rural areas, residents often travel long distances [22,23], so the SCI may indeed be capturing, in part, a person’s likelihood of traveling to another county. This has relevance, of course, to COVID-19 spread.

In particular, the results of this study could be relevant for state and county public health departments in Missouri that are trying to implement COVID-19 prevention practices, such as setting event/business capacity limits or enacting mask requirements. Knowing that social connectedness, as measured through Facebook friendships, is associated with COVID-19 spread even after controlling for the distance between two counties might suggest that mitigation practices should extend beyond a regional approach and be implemented statewide.

Additional investigation is needed to more fully understand the SCI. Our study and others’ prior work have demonstrated a signal, but now more research is needed to fully decipher that signal. We also encourage Facebook to continue to update and refine the SCI, so that researchers can understand more of what in the signal it is capturing and how it relates to COVID-19. However, while that work is underway, there may be utility in using the SCI in models of COVID-19 spread even without knowing what it is capturing. In the case of a global pandemic, the need for timely data and models to inform mitigation efforts is critical. If including the SCI in these models can improve model fit and serve as a control for more fully understood variables, then it is worth including in the model.

### Limitations

There are key caveats that must be acknowledged. First, more granular data are not included in the SCI, which would add greater clarity to the results. For example, we would have liked to have known the demographics of Facebook users in a given county and if the SCI was different for certain demographic subgroups in each county (eg, are older Facebook users in county 1 more likely to form friendships with older users in county 2). Second, the SCI was a cross-sectional data set created in March 2020, while our COVID-19 data were longitudinal from March to September 2020. It is unknown if, and by how much, the SCI changes over time and if this would impact our modeling. Third, we are network analysis and modeling experts; we are not epidemiologists or infectious disease experts. Therefore, we approached this study from a methodological perspective, not a public health perspective, and we acknowledge there are additional factors that should be studied before any policies or prevention practices are enacted based on these results.

### Conclusions

This study further validated the signal raised by the SCI as it relates to COVID-19 spread. It is also the first study to use ERGM to model Facebook friendships as they relate to COVID-19 spread. We found that as the social connectedness increases between two counties, the odds of those two counties having highly correlated COVID-19 case count trajectories increases by 18%, controlling for the counties’ population size, rurality, and the distance between the two counties. This suggests that the SCI is capturing a unique social connection phenomenon that is important in understanding disease transmission and is specifically relevant to COVID-19. Additional research is needed to better understand what the SCI is capturing practically and what it means for public health policies and prevention practices, but in the short term, researchers may consider adding it to other COVID-19 models to improve model fit.

## Appendix

Multimedia Appendix 1 Missouri Counties' COVID-19 Daily Case Counts.

## Abbreviations

AIC: Akaike information criterion
ERGM: exponential random graph model
OR: odds ratio
SCI: Social Connectedness Index
