# Supplementary material for: Utility of Facebook’s Social Connectedness Index in Modeling COVID-19 Spread: Exponential Random Graph Modeling Study
Source: JMIR Public Health Surveill. 2021 Dec 15;7(12):e33617. doi: 10.2196/33617 (PMC8675563; doi:10.2196/33617)
Supplement: Multimedia Appendix 1 [file publichealth_v7i12e33617_app1.docx]

Supplementary File 1 – Missouri Counties’ COVID-19 Daily Case Count Data from March 8, 2020 to September 30, 2020.

| **County** | **Average New Daily Case Count** | **Standard Deviation** | **Minimum** | **Maximum** |
| --- | --- | --- | --- | --- |
| Adair | 1.71 | 2.39 | 0 | 14 |
| Andrew | 1.30 | 2.18 | 0 | 12 |
| Atchison | 0.29 | 0.78 | 0 | 6 |
| Audrain | 2.20 | 4.27 | 0 | 35 |
| Barry | 2.42 | 3.83 | 0 | 21 |
| Barton | 0.94 | 1.74 | 0 | 10 |
| Bates | 0.62 | 1.28 | 0 | 9 |
| Benton | 1.64 | 3.68 | 0 | 26 |
| Bollinger | 1.73 | 4.72 | 0 | 57 |
| Boone | 22.86 | 35.27 | 0 | 190 |
| Buchanan | 9.83 | 13.56 | 0 | 112 |
| Butler | 3.28 | 4.79 | 0 | 27 |
| Caldwell | 0.50 | 1.14 | 0 | 7 |
| Callaway | 3.27 | 5.33 | 0 | 25 |
| Camden | 4.97 | 7.71 | 0 | 50 |
| Cape Girardeau | 8.73 | 12.15 | 0 | 58 |
| Carroll | 0.68 | 1.40 | 0 | 8 |
| Carter | 0.55 | 1.34 | 0 | 7 |
| Cass | 7.41 | 9.60 | 0 | 45 |
| Cedar | 0.60 | 1.39 | 0 | 8 |
| Chariton | 0.28 | 0.64 | 0 | 4 |
| Christian | 7.12 | 11.55 | 0 | 86 |
| Clark | 0.32 | 0.80 | 0 | 5 |
| Clay | 8.87 | 9.29 | 0 | 42 |
| Clinton | 1.25 | 1.98 | 0 | 11 |
| Cole | 7.70 | 11.72 | 0 | 62 |
| Cooper | 2.10 | 3.68 | 0 | 22 |
| Crawford | 2.01 | 3.99 | 0 | 31 |
| Dade | 0.38 | 0.97 | 0 | 6 |
| Dallas | 0.96 | 1.67 | 0 | 9 |
| Daviess | 0.61 | 1.45 | 0 | 9 |
| DeKalb | 0.66 | 1.30 | 0 | 10 |
| Dent | 0.74 | 1.87 | 0 | 12 |
| Douglas | 1.02 | 1.95 | 0 | 12 |
| Dunklin | 3.89 | 5.72 | 0 | 33 |
| Franklin | 8.54 | 10.69 | 0 | 47 |
| Gasconade | 0.90 | 2.43 | 0 | 21 |
| Gentry | 0.66 | 1.44 | 0 | 11 |
| Greene | 30.91 | 44.32 | 0 | 205 |
| Grundy | 0.97 | 2.56 | 0 | 17 |
| Harrison | 0.56 | 1.29 | 0 | 9 |
| Henry | 1.00 | 1.74 | 0 | 11 |
| Hickory | 0.53 | 1.40 | 0 | 11 |
| Holt | 0.39 | 1.67 | 0 | 19 |
| Howard | 0.91 | 1.75 | 0 | 10 |
| Howell | 3.08 | 5.77 | 0 | 32 |
| Iron | 0.40 | 0.97 | 0 | 7 |
| Jackson | 36.85 | 47.54 | 0 | 406 |
| Jasper | 18.56 | 25.22 | 0 | 146 |
| Jefferson | 20.95 | 24.56 | 0 | 98 |
| Johnson | 5.42 | 9.22 | 0 | 56 |
| Kansas City | 54.57 | 55.33 | 0 | 314 |
| Knox | 0.24 | 0.76 | 0 | 5 |
| Laclede | 3.28 | 5.87 | 0 | 47 |
| Lafayette | 2.58 | 4.86 | 0 | 32 |
| Lawrence | 3.07 | 4.78 | 0 | 27 |
| Lewis | 0.47 | 1.12 | 0 | 7 |
| Lincoln | 3.99 | 5.14 | 0 | 26 |
| Linn | 0.50 | 1.21 | 0 | 12 |
| Livingston | 2.00 | 14.44 | 0 | 199 |
| Macon | 0.76 | 1.60 | 0 | 11 |
| Madison | 1.52 | 3.39 | 0 | 23 |
| Maries | 0.43 | 0.92 | 0 | 6 |
| Marion | 3.25 | 5.80 | 0 | 47 |
| McDonald | 5.43 | 15.53 | 0 | 196 |
| Mercer | 0.14 | 0.54 | 0 | 5 |
| Miller | 2.47 | 4.06 | 0 | 16 |
| Mississippi | 1.46 | 2.87 | 0 | 20 |
| Moniteau | 1.67 | 3.08 | 0 | 22 |
| Monroe | 0.44 | 1.00 | 0 | 6 |
| Montgomery | 0.45 | 0.85 | 0 | 6 |
| Morgan | 1.82 | 3.74 | 0 | 24 |
| New Madrid | 2.61 | 4.34 | 0 | 33 |
| Newton | 7.29 | 10.65 | 0 | 88 |
| Nodaway | 3.83 | 10.12 | 0 | 115 |
| Oregon | 0.63 | 1.44 | 0 | 8 |
| Osage | 0.80 | 1.46 | 0 | 9 |
| Ozark | 0.62 | 1.80 | 0 | 18 |
| Pemiscot | 2.59 | 3.41 | 0 | 19 |
| Perry | 3.09 | 4.58 | 0 | 24 |
| Pettis | 5.63 | 8.44 | 0 | 47 |
| Phelps | 2.42 | 4.38 | 0 | 21 |
| Pike | 1.27 | 2.76 | 0 | 20 |
| Platte | 3.44 | 4.34 | 0 | 27 |
| Polk | 3.24 | 7.54 | 0 | 68 |
| Pulaski | 3.38 | 7.03 | 0 | 68 |
| Putnam | 0.16 | 0.44 | 0 | 2 |
| Ralls | 0.75 | 1.62 | 0 | 11 |
| Randolph | 1.32 | 2.61 | 0 | 15 |
| Ray | 0.86 | 1.80 | 0 | 10 |
| Reynolds | 0.23 | 0.65 | 0 | 4 |
| Ripley | 0.86 | 1.72 | 0 | 12 |
| Saline | 3.59 | 4.91 | 0 | 27 |
| Schuyler | 0.13 | 0.44 | 0 | 3 |
| Scotland | 0.17 | 0.45 | 0 | 2 |
| Scott | 4.34 | 5.51 | 0 | 24 |
| Shannon | 0.54 | 1.25 | 0 | 10 |
| Shelby | 0.36 | 1.28 | 0 | 14 |
| St. Charles | 37.47 | 43.05 | 0 | 239 |
| St. Clair | 0.40 | 1.16 | 0 | 10 |
| St. Francois | 10.30 | 20.00 | 0 | 172 |
| St. Louis | 118.71 | 98.02 | 0 | 530 |
| St. Louis City | 35.68 | 23.30 | 0 | 112 |
| Ste. Genevieve | 1.01 | 1.66 | 0 | 8 |
| Stoddard | 2.53 | 3.93 | 0 | 34 |
| Stone | 2.29 | 3.76 | 0 | 23 |
| Sullivan | 1.04 | 2.36 | 0 | 21 |
| Taney | 5.89 | 9.04 | 0 | 70 |
| Texas | 1.61 | 3.86 | 0 | 30 |
| Vernon | 0.94 | 1.73 | 0 | 12 |
| Warren | 2.34 | 3.21 | 0 | 19 |
| Washington | 2.09 | 4.23 | 0 | 30 |
| Wayne | 1.10 | 2.40 | 0 | 14 |
| Webster | 3.22 | 6.38 | 0 | 46 |
| Worth | 0.10 | 0.33 | 0 | 2 |
| Wright | 1.76 | 4.25 | 0 | 28 |
